# Domestication constrains the ability of dogs to convey emotions via facial expressions in comparison to their wolf ancestors

**DOI:** 10.1038/s41598-024-61110-6

**Published:** 2024-05-07

**Authors:** Elana R. Hobkirk, Sean D. Twiss

**Affiliations:** Department of Biosciences, Durham University, Durham, DH1 3LE UK

**Keywords:** Wolves, Domestic dogs, Facial expressions, Emotions, Domestication, Animal welfare, Zoology, Animal behaviour

## Abstract

Dogs (*Canis lupus familiaris*) are the domestically bred descendant of wolves (*Canis lupus*). However, selective breeding has profoundly altered facial morphologies of dogs compared to their wolf ancestors. We demonstrate that these morphological differences limit the abilities of dogs to successfully produce the same affective facial expressions as wolves. We decoded facial movements of captive wolves during social interactions involving nine separate affective states. We used linear discriminant analyses to predict affective states based on combinations of facial movements. The resulting confusion matrix demonstrates that specific combinations of facial movements predict nine distinct affective states in wolves; the first assessment of this many affective facial expressions in wolves. However, comparative analyses with kennelled rescue dogs revealed reduced ability to predict affective states. Critically, there was a very low predictive power for specific affective states, with confusion occurring between negative and positive states, such as Friendly and Fear. We show that the varying facial morphologies of dogs (specifically non-wolf-like morphologies) limit their ability to produce the same range of affective facial expressions as wolves. Confusion among positive and negative states could be detrimental to human–dog interactions, although our analyses also suggest dogs likely use vocalisations to compensate for limitations in facial communication.

## Introduction

Successful communication is essential for highly social, group-living animals as it mediates important social behaviour, upholds social hierarchies and maintains strong social bonds^1–5^. The social communication of mammals has been well studied and includes a vast array of research on visual signalling^6–20^. Moreover, mammalian faces can convey a wealth of information via communicative signals^15,16,18,19^, and as a result, facial expressions are considered highly important for social communication amongst mammals^18^. Conveying information about one’s internal affective state is also essential for social animals as this allows for the selection of appropriate behavioural decisions to be made by receivers, in response to cues from others^21–23^. Affective states are forms of motivation such as emotions and moods^24–33^. The term ‘affect’ is used to describe states that have the property of valence (positive or negative)^30,34^. In non-human animals ‘affective states’ are based on contextualised, behavioural indicators (including body-language, vocalisations, and changes in social proximity) and consist of short-term emotion-like and long-term mood-like states^22,30,34–38^. One method of quantifying affective states is via the movements observed in facial expressions^26,28,29,32,34,39^. As a result, several approaches have been developed to quantify the facial expressions of different species, in particular Facial Action Coding Systems^9,13,39–46^.

**Figure 1:**
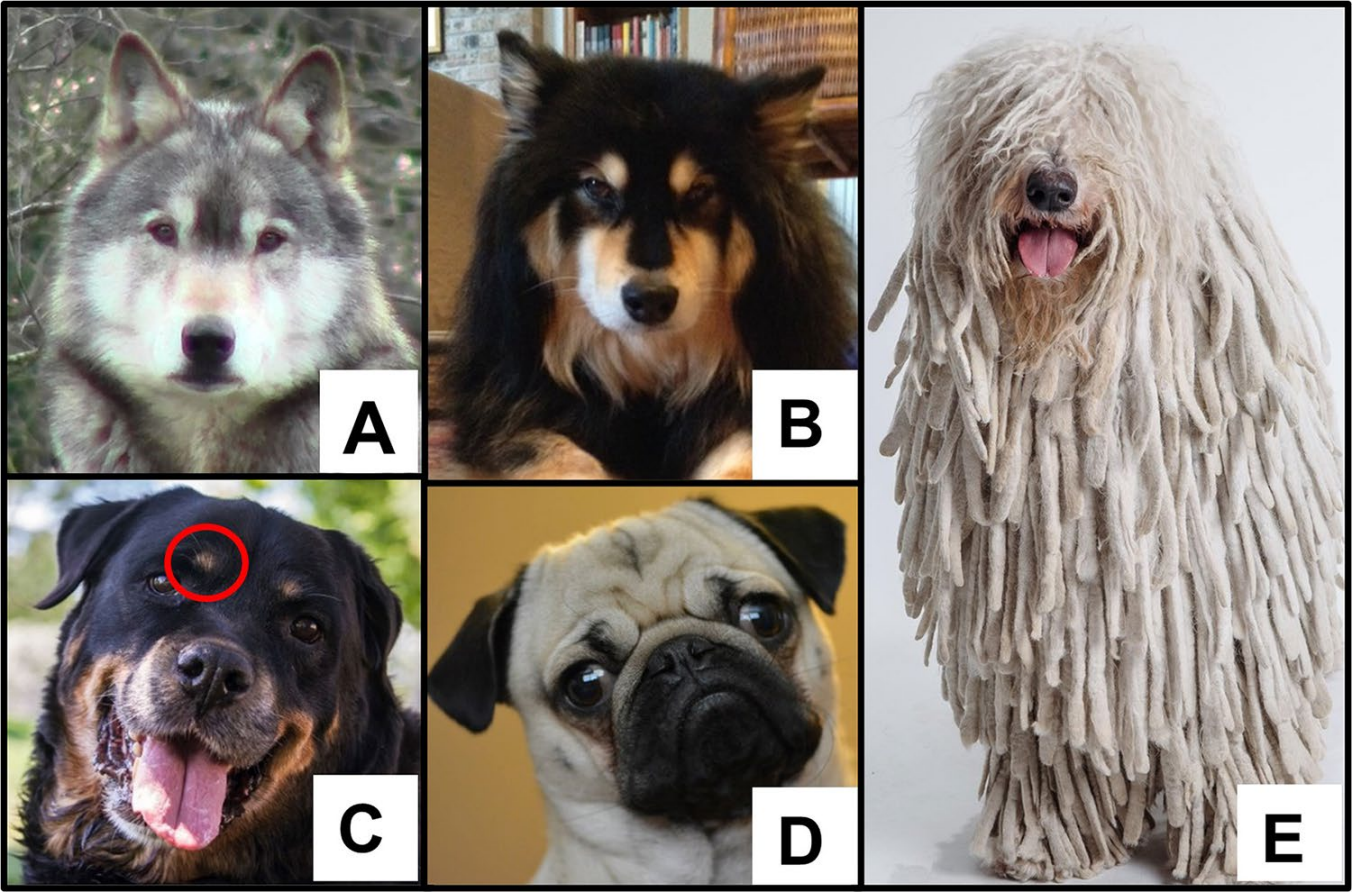
Illustration of the differences in the main conveyers of facial expressiveness between wolf and examples of domestic dog breeds. (**A**) Wolf (*Canis lupus*) portrait depicting typical head morphologies and facial patterning. Note erect ears, head shape, fur length and slope, and facial masking as a consequence of lighter coloured ‘eyebrows’, muzzle and cheek area. Photograph by ER Hobkirk. (**B**) ‘Wolf-like’ Finnish Lapphund dog, with head morphologies and facial patterning almost identical to that of the wolf. Photograph by SD Twiss. (**C**) Typical Rottweiler face with conspicuous brown eyebrows (red circle), set against a solid black background. Note flopped ears and broad head shape in comparison with the wolf. Image courtesy of the American Kennel Club. (**D**) Brachycephalic face of a Pug dog. Note flopped ears, bulging eyes and excessive wrinkling in comparison with the wolf. Image courtesy of the American Kennel Club. (**E**) Komondor dog with less distinct facial features due to fur type (dreadlocks), length and slope. Image courtesy of the American Kennel Club.

One species that has frequently been used as a model for describing the social behaviour of group-living animals is the wolf (*Canis lupus*)^47^, and it has been long speculated that wolves use facial expressions to convey affective states^6,8,48,49^. Schenkel^6^ described more than 20 variations of wolf facial expressions, which he argued were associated with emotion-like affective states. Fox^8^ argued that wolves were capable of a broad range of facial expressions, which are used in varying social interactions and contexts. However, there have been no quantitative analyses of these suggested associations between facial expressions and affective states in wolves. It is thought that the head and facial feature morphologies of wolves aid the production of facial expressions that are key to successful social communication^6,8^. Combinations of facial features, including fur length and fur slope, mimic muscle movements, and the activities of the eyes, nose and ears, emphasise the appearance of the muzzle, lips, eyes, forehead and ears, which are the main conveyors of facial expressiveness^6–8^. The relative shape and position of the main conveyers of facial expressiveness are highly conserved across all wolves throughout the world^50–53^ (Fig. 1A) which highlights the adaptive value of facial communication in wolves. Quantifying these associations between facial expressions and affective states would provide a valuable tool for monitoring welfare in both wild and captive canids and provide the scope for cross-species comparisons to give insight into the evolution and adaptive value of affective states in Canidae.

Like wolves, domestic dogs (*Canis lupus familiaris*) can produce facial expressions^7^ due to their complex facial musculature^7,45,54^ and have been shown to express affective states^55^. However, as a result of selective breeding, head morphologies and the associated main conveyers of facial expressiveness of many breeds of dogs have greatly diverged from those of their wolf ancestors^56–58^ (Fig. 1). While some breeds have retained a more ‘wolf-like’ appearance (Fig. 1B), other breeds differ markedly in head and facial feature morphologies. For example, Rottweillers and Pugs have flopped ears, brachycephalic faces (short, broad skulls and shortened muzzles) and pendulous lips (Fig. 1C and D). The eyes of the Pug are also relatively larger in proportion to the size of its head, and the forehead of the Pug is greatly wrinkled compared to the wolf (Fig. 1A). In addition, the visibility of the main conveyers of facial expressiveness of dogs such as the Komondor is greatly diminished as they are mostly hidden beneath their dreadlock fur (Fig. 1E). To date there has been little quantitative analyses of the associations between domestic dog facial expressions and affective states^21,33,54,55,59^. Therefore, in this study our aim was to first identify discrete facial expression movements within wolves and domestic dogs, and then determine whether combinations of different facial movements correlated with specific affective states. We also predicted that the various head and facial morphologies found across different dog breeds would limit their abilities to successfully produce the range of affective facial expressions observed in wolves. The facial expressions of captive, human-habituated wolves and kennelled rescue dogs were quantified during behavioural events using the Dog Facial Action Coding System (DogFACS)^45^ supplemented with records of Additional Facial Movements (AFM, Table S3). We then tested whether the observed facial expressions mapped onto the affective states exhibited by the wolves and dogs during these behavioural events. Where facial expressions were ambiguously associated with one or more affective state, we investigated the potential reasons for this by comparison with facial morphological differences.

## Results

**Figure 2:**
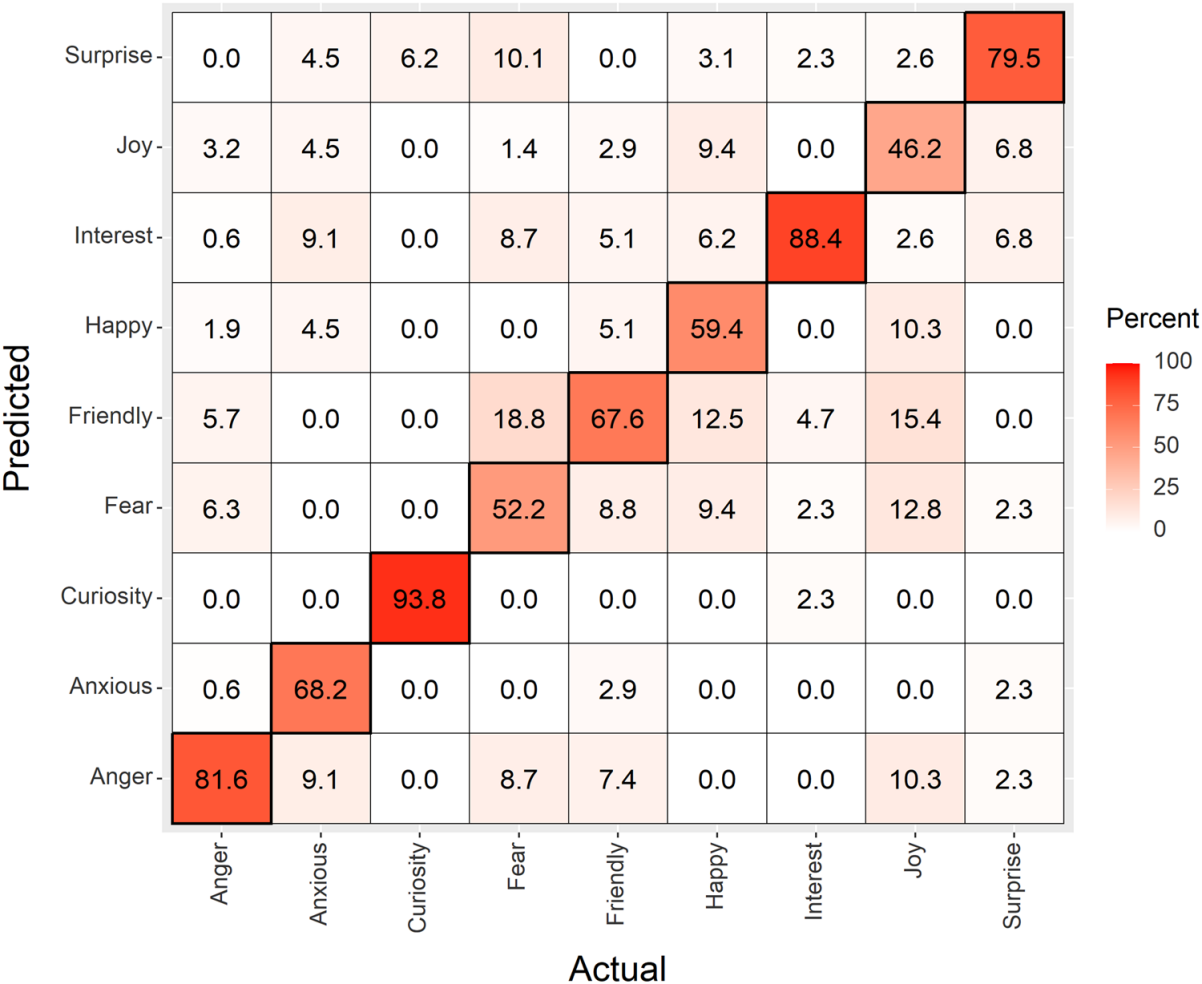
Confusion matrix, showing the observed (actual) versus predicted affective states for wolf facial expressions (*n* = 559). Values within each true positive tile (diagonal) display the precision percentages per affective state. Overall precision = 71%.

We used linear discriminant analysis (LDA) to conduct a supervised classification^60^ of facial movements (DogFACS and AFM codes), to identify how well each directly observed affective state could be predicted based upon the combinations of DogFACS and AFM codes recorded for each event. Predicted wolf affective states based upon LDA of facial movements exhibited substantial agreement with the independently allocated affective state classifications based on direct observation of the videos (71%, Fig. 2). Precision values within individual cells of the confusion matrix range between moderate (lowest precision value for the affective state Joy = 46%) and almost perfect agreement (highest precision; Curiosity = 94%). In addition, where confusion occurs within the matrix (values other than True Positives), the values do not exceed 20% (‘slight agreement’ according to criteria set by Landis & Koch^61^).

**Figure 3:**
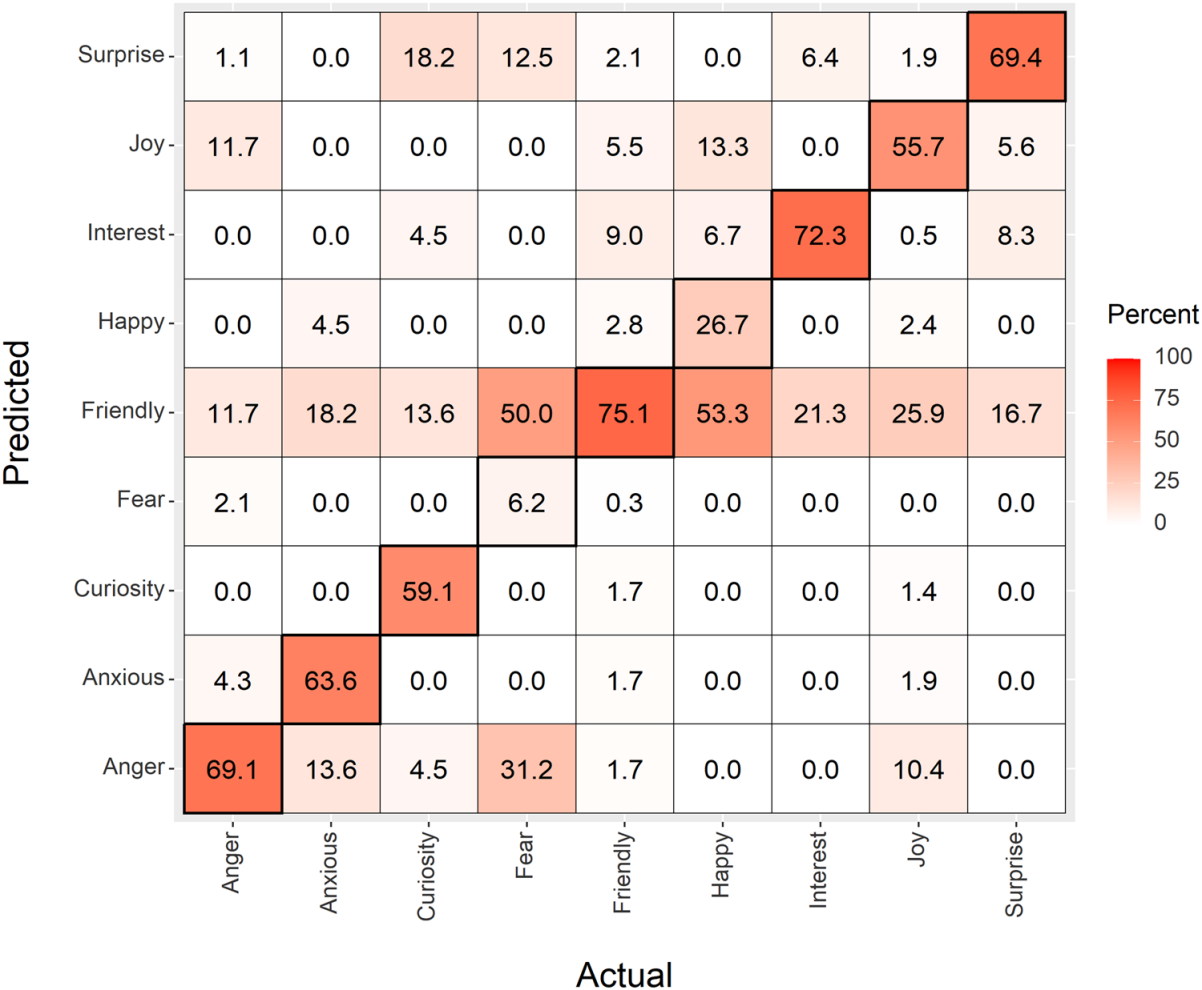
Confusion matrix, showing the observed (actual) versus predicted affective states for dog facial expressions (*n* = 753). Values within each true positive tile (diagonal) display the precision percentages per affective state. Overall precision = 65%.

The confusion matrix for dog affective states (Fig. 3) also shows substantial agreement (albeit lower than that for wolves at 65%) between LDA predicted and observed affective states. However, precision for specific cells within the matrix for dogs is considerably reduced compared to that of wolves, ranging from 6% for the Fear affective state to a maximum of 75% for Friendly. Where confusion occurs, it is typically greater compared to wolves; for example, 31% confusion between Fear and Anger. The affective state termed Friendly has substantial overall precision, with 75% of the events classed as Friendly by both direct observation and the LDA classification. However, 12–53% of the events classed as other affective states (both positive and negative states, Table 1) by direct observation were incorrectly categorised as Friendly based upon the LDA classification of facial movements (Fig. 3). The level of disagreement in Fig. 3 will be dependent upon the particular dogs and breeds included in the analysis, and the range of affective states expressed by each. However, iterated simulations repeating the LDA with each individual (and each breed in further simulations) removed from the analysis in turn revealed no increase in levels of agreement, only more disagreement in some cases (see supplementary material and Table S7a and b). Therefore the 65% agreement in our dog confusion matrix appears to be a maximal estimate.

**Table 1:** Primary, short-term, emotion-like affective states with corresponding descriptors.

Primary affective state and positive (+ ve) or negative (− ve) classifications	Descriptors	Source
Anger (− ve)	Aggressive interactions; can be offensive or defensive; often results in decreased social proximity, unless full conflict occurs	^63–66^
Anxious (− ve)	Focal canid displays signs of distress (e.g. vocalisations such as whimpering), often in response to uncertain anticipation; social proximity is neither increased nor decreased	^64–67^
Curiosity (+ ve)	Focal canid fully approaches ‘emotive’ stimuli (e.g. familiar sounds, such as dog squeak toy) and becomes fixated on it for an extended period (> five seconds)	^64,66,68^
Fear (− ve)	Associated with aggressive interactions and sudden shocks by novel stimuli, e.g. the approach of an unfamiliar social interactant; social proximity is often decreased as focal canid attempts to escape from social interactant or novel stimuli	^63,64,66^
Friendly (+ ve)	Associated with submissive behaviour (e.g. lowered body posture) toward social interactant (who often has a higher social rank); social interactant may be familiar or unfamiliar (human social interactants only); social proximity is increased. This affective state can occur during positive interactions (as active submission) and negative interactions (as passive submission)	^64,66,69^
Happy (+ ve)	Focal canid is receiving tactile attention from social interactant, e.g. grooming or petting; social proximity is increased	^64–66^
Interest (+ ve)	Focal canid approaches social interactant or inanimate object, though makes no attempt to fully interact; social proximity is initially increased but maintained at approximately one body length from social interactant/inanimate object Note: Focal canid may increase or decrease social proximity if social interactant attempts to interact with focal canid	^64,66,70^
Joy (+ ve)	Excitable interactions, e.g. play or copulation; social proximity is increased	^63,64,66,67^
Surprise (+ ve/ − ve)	Focal canid reacts to sudden shocks to the sensory system, in particular auditory, visual and tactile stimuli; focal canid is momentarily fixated on stimuli (< five seconds) and often becomes immobile (‘freezes’); proximity to stimuli is neither increased nor decreased	^63,64,66,71^

Descriptors and positive and negative classifications are derived from contextual information gathered from video footage of canid social interactions and reactions to external stimuli. Source indicates the primary literature for comparison of descriptors and positive and negative classifications. Friendly was classified as positive as focal canids were unharmed during interactions involving this affective state. Surprise was classified as positive or negative.

The confusion seen in Fig. 3 consists of 262 incorrectly predicted affective states out of 753 dog facial expressions analysed. Table 2 shows how these 262 incorrect classifications distribute across dogs with different morphological facial features. Traits that were most associated with confusion within the dog matrix were brachycephalic and mesocephalic (medium proportioned skulls) heads, which together were associated with nearly 80% of the incorrectly predicted affective states, and flopped and semi-flopped ears, which were present in 84% of the incorrectly predicted events (Table 2). Flews (pendulous lips) were present in 40% of the cases of confusion within the dog matrix. Table 2 also shows that dolichocephalic (wolf-like morphology, long length skulls with long muzzles) accounted for 22% of incorrectly predicted affective states, however these dolichocephalic dogs also had non-wolf-like facial morphologies such as flopped and semi-flopped ears.

**Table 2:** Domestic dog morphological facial features and the corresponding number of dogs and percentage of entries within the incorrectly predicted (confused, *n* = 262) affective states seen in Fig. 3 (dog confusion matrix).

Morphological features	*n*, number of dogs	% of entries
Head shape	–	–
Brachycephalic	103	39
Mesocephalic	102	39
Dolichocephalic*	57	22
Ear position	–	–
Flopped	149	57
Semi-flopped	70	27
Erect*	43	16
Face	–	–
Flews	104	40
Ectropion	40	15
Neutral abnormalities	18	7

*wolf-like morphological facial features.

Table 3 shows the frequency and percentage of occurrence for each facial movement per affective state for wolves. Any occurrence of 10% or more was considered to represent movements involved in signalling affective states (10% rule of thumb^61,62^). The affective states of Anxious, Curiosity, Fear, Happy, Interest, Joy and Surprise constitute relatively unique combinations of key facial movements (ranging between four movements for Joy and 12 movements for Fear) with little overlap, whereas Anger and Friendly constitute a wide range of facial movements (29 for Anger and 27 for Friendly) with a large degree of overlap. However, despite this overlap key differences still exist. These include the absence of AU118 (Fisher’s exact test *p* < 0.001), EAD101 (Fisher’s exact test *p* < 0.001), EAD102 (Fisher’s exact test *p* = 0.07), and JSNAP (Fisher’s exact test *p* < 0.001) for Friendly, and the absence of AD55 (Fisher’s exact test *p* = 0.00) and AD40 (Fisher’s exact test *p* = 0.06) for Anger.

**Table 3:**
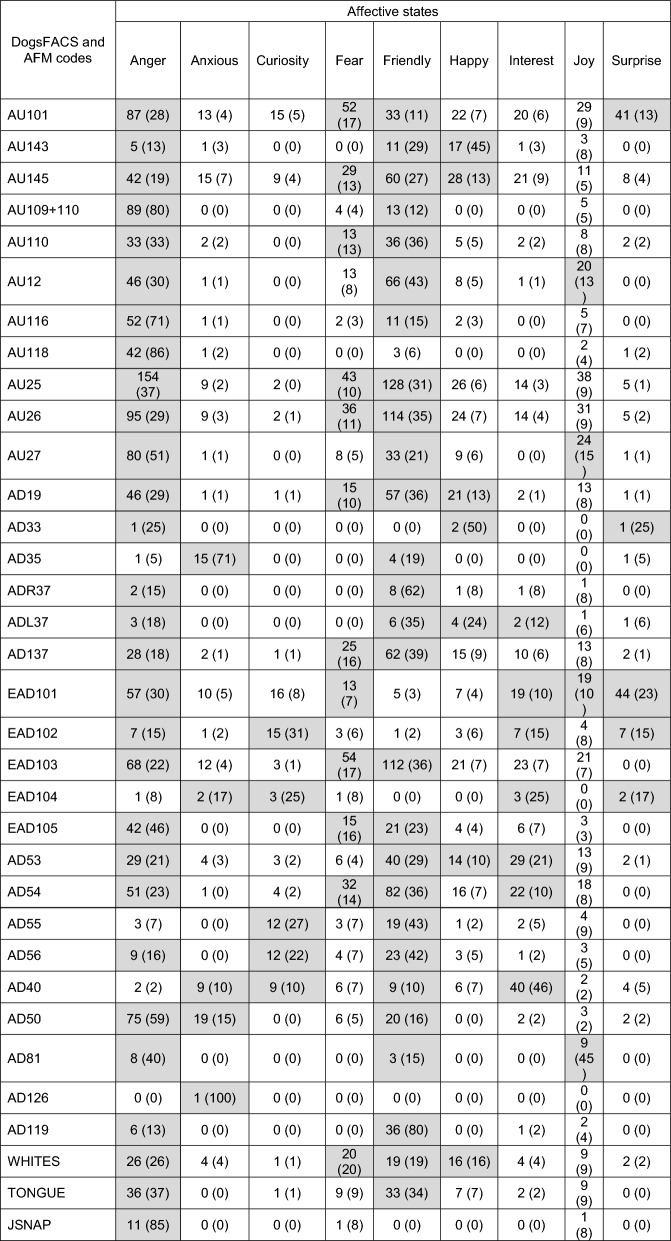
Frequency and percentage (in brackets) of occurrence of DogFACS and Additional Facial Movement (AFM) codes per wolf affective state.

Accepted percentages (10% and above^62^,) are highlighted in grey, *n* = 559.

Table 4 shows the frequency and percentage of occurrence for each facial movement per affective state for domestic dogs. Using the 10% rule of thumb^61,62^ Anger and Friendly comprise the greatest range of facial movements, similar to the patterns observed for wolves in Table 3. However, for dogs Joy also encompasses a wide range of facial movements, in fact all but one facial movement (AD119, licking) are observed in events associated with Joy. Table 4 shows extensive overlap of facial movements between Anger, Friendly and Joy. Conversely, there are no facial movements that attain the 10% threshold for the affective state of Fear, and the remaining affective states comprise very few facial movements that meet the threshold, ranging between just one (for Happy and Interest) and three (for Curiosity and Surprise). In addition, Table 4 illustrates the 55 similarities (blue numbers) and 82 differences (red numbers) in the use of facial movements by dogs compared to wolves. This suggests that domestic dogs only in part, produce facial expressions like wolves, and do so for a limited range of affective states.

**Table 4:**
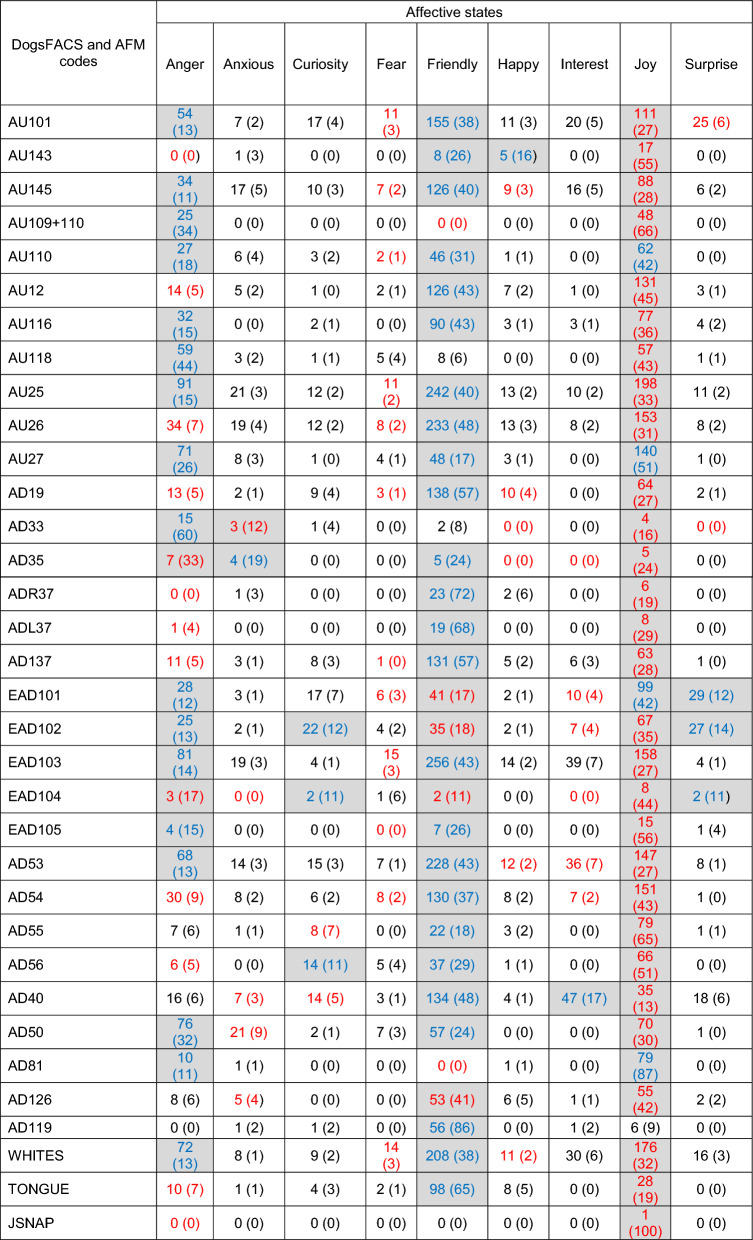
Frequency and percentage (in brackets) of occurrence of DogFACS and Additional Facial Movement (AFM) codes per domestic dog affective state.

Accepted percentages (10% and above^62^), are highlighted in grey, Blue numbers represent similarities between dogs and wolves (Table 3) in the use of facial movements for each affective state. Red numbers represent differences of dogs and wolves in the use of facial movements for each affective state. *n* = 753. See Table S2 for breed-type information.

Although our analysis focused on visual signalling, throughout our video decoding we also noted when vocalisations (AD50, Table S3) occurred during events. It was found that dogs vocalise more often than wolves when socially interacting and reacting to ‘emotive’ stimuli (dogs; n = 298/753 (40%), wolves; n = 137/559 (25%); X^2^ = 32.9,* p* < 0.00001). This increased use in vocalisations suggests that auditory communication may be a more important aspect of communication for conspecific interactions among domestic dogs compared to interaction between wolves. Within the events that comprise the correctly predicted affective states of Fig. 3 (*n* = 491) dogs were found to use vocalisations 29% of the time. However, vocalisation use increased to 35% within the events that were not classified correctly according to the predicted affective state (*n* = 262). The various types of vocalisations used in the context of different affective states for dogs and wolves are detailed in the supplementary material (Tables S4, S5 and S6).

## Discussion

Here we show that distinct combinations of facial movements relate to specific affective states in wolves and dogs. However, while wolf affective states can be identified very well via facial movements, there is less clarity among domestic dogs. Our results show a greater degree of confusion within the dog matrix with reduced overall precision compared to that for wolves. The precise level of disagreement between actual and predicted affective states will be dependent upon the particular dogs and breeds included in an analysis of this type, although our simulations indicate that our estimate of the extent of confusion within the dog matrix is in fact quite conservative. However, it is not the reduced overall agreement that is the most critical difference between wolves and dogs, but rather, where the confusion exists within the matrices that is most revealing. Overall, these data suggest that dogs are limited in their ability to produce facial expressions for a wide range of affective states.

Domestication has resulted in the morphologies of many species diverging greatly from that of their wild, ancestral counterparts^72,73^. As a result, domesticated mammals generally possess a range of morphological traits that visibly distinguish them from their wild counterparts, in particular elements of head allometry^72,73^. The divergent head and facial feature morphologies of some domestic dog breeds likely constrain their ability to produce facial expressions that unambiguously convey specific affective states, with head shape and ear position being the most limiting. The brachycephalic and mesocephalic head morphologies seen in many dog breeds have muzzles that are proportionally shorter relative to the overall skull length than those of wolves (dolichocephalic heads), and therefore have facial features that are compacted together^74^. Movements involving the muzzle, nose, lips and tongue appear important for the successful production of affective facial expressions in wolves, but these movements are less frequently observed in dogs. For example, wolves use the facial movement ‘nose wrinkler and upper lip raiser’ (AU109 + 110) 80% of the time in the context of Anger (89 out of 111 events, Table 3), but domestic dogs (across breed-types) only use this movement 34% of time in the same context (25 out of 73 events, Table 4). Similarly, wolves sniff (AD40) 46% of the time in the context of Interest (40 out of 87 events, Table 3), but dogs sniff only 17% of the time in the context of Interest (47 out of 278 events, Table 4). The mimic muscles, especially those around the muzzle, lips and nose of brachycephalic and mesocephalic dogs, have much less space to develop to the same size as those seen in dolichocephalic heads. Therefore, some muscles are likely too small to produce the full range of movements needed to produce appropriate facial expressions.

It should be noted that the mimic muscles of wolves and dogs are the same except for the levator anguli oculi medialis muscle (LAOM). Kaminski et al.^54^ found that domestic dogs raise their inner brows (AU101) more frequently and with higher intensity than wolves do due to the presence of a larger, ‘fully developed’ LAOM in dogs compared to wolves. Kaminski et al.^54^ suggest that the ‘fully developed’ LAOM in dogs is the result of the necessity for dogs to communicate with humans. However, wolf-human interactions are unlikely to represent a context in which wolves are likely to use AU101. Wolves do not interact with humans in the same way that dogs do, and unlike dogs, wolves essentially perceive humans as heterospecifics^75–81^. The LAOM of wolves is comprised of relatively limited muscle tissue and a tendon, which Kaminski et al.^54^ claim produced the low frequency and low intensity of AU101 seen in wolves. While we do not dispute that an ‘under-developed’ LAOM likely results in decreased intensity of AU101, we do argue that the presence of a tendon with relatively limited muscle instead of the ‘fully developed’ LAOM that Kaminski et al.^54^ observed in some dog breeds (5 out of 6 breeds examined) may provide adequate, frequent movement of AU101 in wolves as tendons can enhance muscle performance^82^. Moreover, it is suggested that wolves see the world ‘faster’ than humans do, in that they have a greater sensitivity to motion, and are therefore able to make finer temporal use of visual information^52^. If wolves see the world ‘faster’, we argue that their use of AU101 is subtle, yet adequate for communication within wolf–wolf social interactions. Analysis of wolf facial movements requires the use of slow-motion video footage to fully allow movements to be detected by humans. By contrast, dogs exhibit a more exaggerated use of AU101, hence the larger, ’fully developed’ LAOM, allowing humans to detect the movement with the naked eye in real-time. While Kaminski et al.^54^ highlight a fascinating response to domestication with regards to the importance of AU101 for dog–human communication, their findings unfortunately do not directly address the importance of AU101 in wolf-wolf social communication.

In addition to head shape and muzzle length limitations, flopped and (to a lesser degree) semi-flopped ears were also associated with much of the confusion seen within the dog matrix. Ear movements appear to be important for the production of affective facial expressions in wolves (Table 3), but again there is a reduction in the use of ear movements across dog breed-types (Table 4). For example, wolves use the movement ‘ears forward’ (EAD101) 30% of the time in the context of Anger (57 out of 190 events, Table 3), while dogs use the same ear movement 12% of the time in the context of Anger (28 out of 235 events, Table 4). Similarly, wolves use the movement ‘ears adductor’ (EAD102) 31% of the time in the context of Curiosity (15 out of 48 events, Table 3), and again dogs only use this movement 12% in the same context (22 out of 191 events, Table 4). Waller et al.^45^ reported that only dogs with erect (wolf-like) ears could produce the DogFACS movement ‘ears rotator’ (EAD104), which demonstrates that departure from wolf-like head and facial feature morphologies can impair the ability of dogs to produce certain facial movements, which is reflected in the reductions of ear movements used by dogs in this study.

Flews (pendulous lips) seem to contribute to much of the confusion within the dog matrix, which is likely due to flews reducing the visibility of some facial movements. For example, Waller et al.^45^ reported that flews reduced the visibility of the facial movement ‘jaw drop’ (AU26), and we observed that the movement ‘tongue show’ (AD19) was difficult to discern in dogs with flews, which is reflected in the results. For example, jaw drop is used by wolves in the context Anger and Fear, 29% (95 out of 330 events) and 11% (36 out of 330 events) of the time (respectively, Table 3), but across dog breed-types this movement is only observed 7% of the time (34 out of 488 events) in the context of Anger, and a mere 2% of the time (8 out of 488 events) in the context of Fear (Table 4). Tongue show is used by wolves in the context of Anger 29% of the time (46 out of 157 events, Table 3), but dogs used this movement only 5% of the time in the same context (13 out of 241 events, Table 4). Therefore, these findings suggests that dogs are not just limited in their range of facial expressions due to smaller, compacted muscles (as seen with brachycephalic and mesocephalic dogs), but they are also limited due to exaggerated facial features such as long flopped ears and flews that obscure the use of mimic muscles and therefore, facial movements.

Neutral abnormalities (deformations of the main conveyers of facial expressiveness) and ectropion (drooping eyelids) contributed to only a small percentage of confusion within the dog matrix. However, few dogs in this study had neutral abnormalities (*n* = 6), which means their effect on the results when compared to all other dogs in this research is minimal. The inclusion of more dogs with neutral abnormalities would give a better indication whether such facial features impede ability to successfully produce consistent affective facial expressions. Ectropion causes the constant exposure of the whites of the eyes (sclera), and the DogFACS system uses the exposure of the sclera to determine eye movements. Eye movements were not analysed in this research, therefore, we are unable to comment directly on how ectropion affects the successful production of affective facial expressions in domestic dogs. However, our results do show that in the context of Friendly and Joy, dogs (across breed-types) expose their sclera (WHITES, Table S3) more than wolves (Tables 3 and 4), which suggests ectropion does indeed impede the production of affective facial expressions in domestic dogs.

The varying head and facial feature morphologies of dogs (in particular, non-wolf-like morphologies) limit their ability to produce the same range of affective facial expressions as their wolf ancestors. However, our research also provides preliminary evidence that domestic dogs may compensate for their limited range in affective facial expressions by using vocalisations. Our analysis of vocalisations found that dogs vocalise more often than wolves when socially interacting and reacting to ‘emotive’ stimuli, suggesting that dogs with limiting facial morphologies may compensate by using more vocalisations to convey their affective states. Certainly, domestic dogs are known to be very vocal in comparison to wolves and will bark for a range of reasons, such as play, defence, threat, pain and loneliness^83,84^. It has been argued that dog vocalisations have been artificially selected for the purpose of inter-specific communication with humans (for working purposes)^84^. However, our results raise the possibility that domestic dogs may use vocalisations for intra-specific communication where facial expressions are not adequate.

In addition to morphological constraints on facial expression, there are other factors that may reduce the ability of domestic dogs to convey affective states via facial expressions. For example, kennel environments can impact on the behaviour of domestic dogs^85–87^ especially, kennel environments that lack housing of dogs in groups, have a lack of dog–human contact and a lack of enrichment (such as toys)^85,86^. However, most of the dogs we used were housed together in groups, they were provided with regular human contact (in preparation for adoption) and they were provided with enrichment in the form of toys. Although behavioural data derived from studies of shelter dogs should be considered with caution due to potential for aberrant behaviour patterns, here we minimised this risk by selecting only individuals deemed suitable for adoption. Nevertheless, a kennel environment can never fully mimic a human home environment that domestic dogs generally live in. Therefore, a comparison of facial expressions across affective states in both kennel and home environments should be considered in future work. Likewise, the past histories of the dogs used are unknown, therefore it is possible the ontogenetic process of enculturation of the dogs used may have resulted in some dogs learning atypical social signalling^88–90^. Therefore, knowing the past histories of the dogs used would be beneficial to help explain unusual findings or indeed, allow one to select dogs that are well-versed in their social abilities and who display typical behaviours. Raising domestic dogs from puppies to adults would allow their past histories to be fully studied and documented and this should be considered for future work.

A key outcome from our study is that we observed considerable confusion between positive and negative affective states for domestic dogs, a pattern that was not seen in our analyses of wolf facial expressions. For example, among dogs, 50% of events allocated an affective state of Fear by direct observation, were classed as Friendly based upon LDA of facial movements. This implies that domestic dogs (across breed-types) are inconsistent in the way they convey affective states via facial expressions. Such high levels of confusion between positive and negative affective states is potentially detrimental to dog–dog and dog–human communication^91^. For example, many dogs that are fearful can become ‘fear aggressive’ and will bite to defend themselves from potential threats^92,93^. If a dog or human was to mistakenly perceive that another dog was displaying a Friendly affective state, when in fact it was displaying Fear, this may lead to dog–dog conflict, or the human being bitten. Therefore, it is important for dog welfare and dog bite prevention for humans to correctly identify the affective states of dogs.

## Conclusion

We have provided the first quantitative evidence that shows distinct combinations of facial movements relate to specific affective states in wolves. Further, we show that divergent head and facial feature morphologies among dog breeds can impair their ability to produce affective facial expressions relative to their wolf ancestors. It is well known that selective breeding has led to a wealth of physical health problems in many domestic dog breeds^94–101^. However, here we provide evidence that such selective breeding also generates social communicative limitations in domestic dogs.

## Methods

### Study sites and subjects

Observations were conducted at two facilities; The UK Wolf Conservation Trust (UKWCT, Beenham UK, 51.42 N, − 1.15 W) and Dogs Trust Darlington (Sadberge UK, 54.56 N, − 1.47 W). Observations at the UKWCT were conducted between February 15th 2016 and March 4th 2016, on weekdays between 0900 and 1700 h (GMT), amounting to 15 days in total. Observations were conducted at Dogs Trust Darlington between August 9th 2016 and November 11th 2016, on weekdays between 1100 and 1700 h (BST), amounting to 21 days in total. The UKWCT provided 10 wolves, five female and five male, which included different sub-species (details in supplementary information Table S1) that were habituated to the presence of humans. Dogs Trust Darlington provided 64 domestic dogs; 43 standard-breeds and 21 cross-breeds (details in supplementary information Table S2). All dogs were adults and consisted of both females (*n* = 21) and males (*n* = 43). Wolves and dogs were housed in small packs of two to three individuals and were free to roam about their enclosures and interact with pack mates and humans during data collection. All dogs used in this study were individuals assessed as suitable for adoption by the Dogs Trust, and no data were collected from dogs deemed to exhibit behavioural issues.

### Video collection

Video clips of wolves (*n* = 559) and dogs (*n* = 753) engaged in spontaneous social interactions or reactions to external ‘emotive’ stimuli (both referred to as ‘events’) were recorded ad-hoc using a hand-held Canon Legria HFR36-D video camera (51 × zoom). Average duration of each event was < 10 s. Social interactions commenced when eye-contact was made between a focal canid and one or more social interactants, with the focal canid becoming immediately focussed upon the interactant(s). Interactions ceased when the focal canid and social interactant(s) dispersed, and eye-contact was lost. External stimuli consisted of easily identifiable auditory and visual stimuli, specifically wind generated sounds, overhead aircraft, and novel objects placed around the study sites (by staff for various public engagement events). Other external stimuli were created using pre-recorded sounds randomly played to wolves and dogs by ERH, on a Nokia Lumia 820 mobile phone. Sounds used were one of four naturally occurring animal vocalisations (rabbit distress call, fawn distress call, squirrel alarm call, domestic dog puppy whines) and one unnatural sound (dog squeak toy). Reactions to stimuli were analysed when a focal canid reacted immediately (within less than one second) to an external stimulus and became focussed upon the direction of the stimulus origin (the focal canid attempted to make eye-contact with the stimulus). The end of an event was defined by the cessation of the external stimulus and the focal canid averting its gaze from the stimulus origin.

### Assessment of affective state for each event

For each event ERH identified a single primary, short-term, emotion-like affective state (Table 1) based on subjective appraisal of key descriptors (sound stimuli were used to specifically evoke Curiosity, Interest and Surprise affective states). Each of these primary affective states are described in previous literature and have been observed in non-human animals^6,21,49,52,63,65,67,68,70,102–110^. However, the descriptions of these primary affective states vary among authors. Therefore, we produced descriptors (derived from contextual information of the video footage obtained, Table 1) to identify the motivation (the functional response) of the focal canid for each event. The affective state Friendly was also included as this affective state has been qualitatively described, but not quantified, in wolves and dogs^52,69,104,111^. In addition, the ability to identify and assess both positive and negative affective states in non-human animals is necessary to fully evaluate the psychological well-being and health of an individual animal^112^. Therefore, each primary affective state investigated in this research was also classified as either positive or negative (Table 1). Although the affective state Friendly can be classified as positive or negative^69,71^, for the purposes of this research, Friendly was categorised as positive as focal canids were unharmed during interactions involving this affective state (Table 1). The affective state Surprise was classified as both positive or negative (Table 1)^71^. The reliability of affective state classifications was independently verified. Seven independent observers were tasked to identify the primary affective state in a random sample of video clips representing one example of each of the affective states identified in both wolf and dog events (amounting to nine dog interactions and nine wolf interactions in total). Inter-observer concordance analyses^113–116^ of affective states was performed using the R^117^ package ‘raters’^118^, showing substantial inter-rater agreement at 70% (according to the Kappa statistic^61^) for all affective states. Inter-observer concordance analyses of positive and negative classifications of affective states showed almost perfect agreement at 82% (according to the Kappa statistic^61^).

### Quantification of facial expressions observed during events

DogFACS^45^ was used to decode video footage of wolf and dog facial expressions. DogFACS comprises a list of 43 codes (see www.animalfacs.com, and supplementary material Table S3) that correspond to specific facial landmarks that move in association with the underlying mimic muscles^45^. We also noted the occurrence of three Additional Facial Movements (AFM); jaw snapping, tongue flicking, and ‘whites of eyes visible’ (see supplementary material Table S3 for full definitions). Jaw snapping and tongue flicking involve movement of the face and have been reported to occur in wolves^8,52^, therefore they were included in our analyses. ‘Whites of eyes visible’ was included as sclera visibility during facial communication was observed frequently in our videos, suggesting importance for communication^45,119,120^. All videos were decoded by ERH (certified DogFACS coder) in slow motion (0.25 × playback speed, using AVS video editor 7.2.), which allowed both obvious and very subtle facial movements to be detected. Although developed for use with domestic dogs, DogFACS was used also to decode wolf facial expressions on the basis that the mimic muscles of wolves and dogs are the same except for the levator anguli oculi medialis muscle (LAOM) being more ‘fully developed’ in dogs compared to wolves^54^. All DogFACS codes that occurred within a single facial expression were recorded once as either ‘on’ or ‘off’ (producing binary data). This method was employed to objectively record the range of facial movements in each facial expression, allowing individual wolf/dog facial expressions to be quantified. This yielded a database of all facial movements observed during each event. For the DogFACS and AFM coding ERH conducted intra-rater reliability coding of 20% of the original video footage processed (263 video clips) one year after her original coding^54^. Using Wexler’s agreement, overall intra-rater reliability resulted in almost perfect agreement at 98%^13,45^.

Previous studies coding facial expressions using DogFACS have not exceeded the use of more than one or two codes per facial expression^45,54,55^. Here we use all relevant codes with the following exceptions. During events canids would alter their gaze and head orientation (in the sagittal plane) only to maintain eye contact with social interactants and reaction stimuli, consequently, all DogFACS eye movements and left and right head movements were removed from subsequent data analyses. DogFACS ‘Body shake’ was also removed from data analyses as this code is predominantly a body movement with associated involuntary head and facial movements. In addition, DogFACS ‘other’ Action Descriptors (ADs) are associated with the visibility of focal canid faces in video footage, and not with facial movements. Therefore, ‘other’ ADs were removed from analyses, as videos lacking canid face visibility were not decoded.

### Testing whether specific combinations of facial action codes predict affective states

We employed linear discriminant analysis (LDA)^60^ with leave-one-out cross-validation using the R package ‘MASS’^121^ to conduct a supervised classification of the DogFACS and AFM data, to identify how well each affective state could be predicted based upon the combinations of DogFACS and AFM codes recorded for each event. We produced separate confusion matrices for the wolf and dog data to examine how well combinations of facial movements map onto the identified affective states^60^. Overall precision (positive predictive value: ratio of correct positive predictions to total predicted positives) of the confusion matrices provided a measure of the general agreement between observed affective state and predictions based on facial movements for wolves and dogs^61^. Examination of individual cells within the matrices provided insights into which affective states were poorly defined by specific combinations of facial expressions, and which were well defined.

The level of agreement within matrix cells was categorised according to Cohen’s kappa^61,113–116^ using the R package ‘raters’. Levels of agreement were categorised according to Landis & Koch (1977; < 0% = Poor, 0–20% = Slight, 21–40% = Fair, 41–60% = Moderate, 61–80% = Substantial, 81–100% = Almost perfect)^61^. Where confusion did occur in the matrix for dogs, events that were incorrectly allocated to affective states were examined in more detail. The morphologies of the dogs involved in these incorrect classifications were tabulated to identify potential links between morphological divergences (from wolves) and the inability to classify affective states based upon facial expressions. Finally, using a 10% rule of thumb as a measure of acceptance^61,62^, the DogFACS and AFM codes per affective state were examined to determine which codes pertained to each affective facial expression for both wolves and dogs.

The level of disagreement in our confusion matrix for dog affective states will to some extent be dependent upon the particular breeds of dog and individuals used, and the range of affective states expressed by each. Therefore, to examine the effect of individual and breed on the levels of disagreement between actual and predicted affective state in our confusion matrix for dogs we conducted the following simulations. We iteratively re-ran our linear discriminant analysis (LDA) with the same data but removing each individual in turn. We then examined the overall level of agreement between actual and predicted affective state allocations in the resulting confusion matrices in comparison to the level of agreement reported for our LDA utilising the full data set.

### Significance

We demonstrate that identifiable combinations of facial movements relate to nine specific affective states in wolves, whereas divergent head and facial feature morphologies among domestic dog breeds limit their ability to produce the same affective facial expressions. It is well known that selective breeding has led to a wealth of physical health problems in many domestic dog breeds. Here we show that selective breeding also generates social communicative limitations in dogs, potentially impacting dog–human interactions. Quantifying associations between facial expressions and affective states may provide a foundation for monitoring welfare in wild and captive canids and allows for cross-species comparisons to yield insight into the emotional evolution in Canidae.

### Ethical approval

All data collection consisted of non-invasive behavioural observations at the UK Wolf Conservation Trust (UKWCT) and Dogs Trust Darlington, approval for data collection was granted by Dogs Trust and UKWCT. All observational protocols were approved by Durham University’s Animal Welfare Ethical Review Board (AWERB), and all procedures complied with BIAZA and Dogs Trust ethical guidelines.

### Supplementary Information

Supplementary Information 1.

Supplementary Information 2.

Supplementary Information 3.

Supplementary Information 4.

Supplementary Information 5.

## Data Availability

Data and code are provided as electronic supplementary material.
