# Supplementary material for: Domestication constrains the ability of dogs to convey emotions via facial expressions in comparison to their wolf ancestors
Source: Sci Rep. 2024 May 7;14:10491. doi: 10.1038/s41598-024-61110-6 (PMC11076640; doi:10.1038/s41598-024-61110-6)
Supplement: Supplementary file 4 — Supplementary Information 4. [file 41598_2024_61110_MOESM4_ESM.pdf]

Supplementary material

Title: Domestication constrains the ability of dogs to convey emotions via facial expressions in comparison to their wolf ancestors.

Authors:

Elana R. Hobkirk[1*] & Sean D. Twiss [1]

Details of study animals.

The UKWCT provided 10 wolves (which included different sub-species, Table S1) that were habituated to the presence of humans. Dogs Trust Darlington provided 64 domestic dogs; 43 standard-breeds (recognised by the UK and American Kennel Clubs), and 21 cross-breeds (including six Lurchers, Table S2). All dogs were adults and consisted of both females (n = 21) and males (n = 43). Wolves and dogs were housed in small packs of two to three individuals and were free to roam about their enclosures and interact with pack mates and humans during data collection.

Table S1. Wolves observed for this research at the UK Wolf Conservation Trust wolf.

Wolf ID	Sex	Age (years)	Species classification
Mai*	Female	9	*Canis lupus*
Matomo*	Male	7	*Canis lupus*
Mosi	Female	9	*Canis lupus*
Torak	Male	9	*Canis lupus* X *Canis lupus lupus* (hybrid)
Sikko	Female	4	*Canis lupus arctos*
Massak	Male	4	*Canis lupus arctos*
Pukak	Male	4	*Canis lupus arctos*
Tundra	Female	4	*Canis lupus*
Tala	Female	4	*Canis lupus*
Nuka	Male	4	*Canis lupus*

Table S2. Domestic dogs observed for this research at Dogs Trust Darlington. Typical adult body sizes (bold text, in descending order) are shown per 'breed-type', be that standard-breed (according to UK and American Kennel Club standards) or cross-breed.

**Breed-type and body size**	*n*, Number of dogs		
	**Females**	**Males**	**Total**
**Giant**			
American Bulldog	1	0	1
**Large**			
Akita	0	1	1
German Shepherd Dog	0	2	2
Greyhound	0	2	2
Labrador Retriever	4	2	6
Boxer Dog	0	3	3
**Medium**			
German Shorthaired Pointer	0	1	1
Siberian Husky	1	0	1
Saluki	0	1	1
Basset Hound	0	1	1
English Springer Spaniel	0	1	1
Border Collie	1	3	4
English Cocker Spaniel	0	2	2
Whippet	0	2	2
**Small**			
Staffordshire Bull Terrier	3	2	5
Dachshund	0	1	1
Beagle	1	0	1
Jack Russel	2	3	5
Lhasa Apso	0	1	1
Shih Tzu	0	1	1
Japanese Chin	0	1	1
**Mixed**			
Lurcher**	1	5	6
Cross-breeds	6	8	15

** Explicit cross-breeds, typically of a sighthound crossed with another breed.

Table S3. The Dog Facial Action Coding System (DogFACS) codes and corresponding names for quantifying specific facial movements in domestic dogs (adapted from Waller et al. 2013) and Additional Facial Movement (AFM) codes recorded in this research.

**Action Units (AUs)**	**Name of movement**	**ADs continued**	**Name of movement**
AU0	No movement	**Head**	
**Upper face**		AD51	Head Turn Left
AU101	Inner brow raiser	AD52	Head Turn Right
AU143	Eye closure	AD53	Head Up
AU145	Blink	AD54	Head Down
**Lower face**		AD55	Head Tilt Left
AU109+110	Nose wrinkler and upper lip raiser	AD56	Head Tilt Right
AU110	Upper lip raiser	**Eyes**	
AU12	Lip corner puller	AD61	Eyes Turn Left
AU116	Lower lip depressor	AD62	Eyes Turn Right
AU118	Lip pucker	AD63	Eyes Up
AU25	Lips part	AD64	Eyes Down
AU26	Jaw drop	**Gross behaviour ADs**	
AU27	Mouth stretch	AD40	Sniff
**Ear Action Descriptors (EADs)**		AD50	Vocalisations
EAD101	Ears Forward	AD81	Chewing
EAD102	Ears Adductor	AD126	Panting
EAD103	Ears Flattener	AD119	Lick
EAD104	Ears Rotator	AD160	Body Shake
EAD105	Ears Downward	**Other ADs**	
**Action Descriptors (ADs)**		AD70	Frontal Region Not Visible
**Muzzle**		AD71	Eyes Not Visible
AD19	Tongue Show	AD72	Lower Face Not Visible
AD33	Blow	AD73	Entire Face Not Visible
AD35	Suck		
AD37	Lip Wipe		
AD137	Nose Lick		
**Additional Facial Movements (AFM) codes**			
JSNAP	'jaw snapping'; non-vocal auditory communication, resulting from the sudden, rapid closing of the jaws of a focal canid.		
TONGUE	'Tongue flicking'; focal canid protrudes its tongue from its mouth, and immediately retracts tongue back into the mouth without licking the lips or nose, a social interactant or an inanimate object.		
WHITES	'Whites of eyes visible'; when the whites (sclera) of a canids eye(s) become clearly visible.		

Table S4. Vocalisations observed for wolves and domestic dogs during data collection, with corresponding descriptors, including frequently observed DogFACS codes for each vocal.

**Vocals**	**Canid**	**Descriptors**
Bark	Wolf & Dog	Explosive, short, sharp, deep cry. Mouth is stretched (AU27) wide to achieve vocal.
Chuff	Wolf & Dog	Short, low sounding 'chuff' sound created by the rapid expulsion of air through the lips (AD33). Mouth is usually closed, or only partially opens via a jaw drop (AU26).
Growl	Wolf & Dog	Long, low guttural rumbling. Mouth is often closed, or partially open (AU26).
Growl-Bark	Wolf & Dog	Long, low rumbling followed immediately by explosive, sharp cry. Mouth often closed initially, then opens wide (AU27) towards end of vocal.
Grunt	Dog	Short, low, guttural vocal originating from the throat. Mouth often partially open (AU26).
Howl	Wolf	Long, doleful, drawn-out cry. Mouth opens wide (AU27) with puckering (AU118).
Whimper	Wolf & Dog	Short, high-pitched, repetitive calls. Air appears to be sucked in through lips (AD35) with mouth closed.
Whine	Wolf & Dog	Long, high-pitched, repetitive calls. Mouth initially closed, but often opens (AU27) between whines.
Yelp	Wolf	Short, sharp, high-pitched cry. Mouth opens wide (AU27).
Yelp-Bark	Wolf	Initial short, sharp high-pitched cry followed immediately by short, sharp deep cry. Mouth is wide (AU27).

Vocalisations occurring during observed events were categorised as detailed in Table S4. Tables S5 and S6 provide the cumulative frequencies (and percentages) of vocalisations by type for each affective state. Table S5 shows that wolves use bark vocalisations in the context of Anxious (67% of events in which vocalisations occurred) and Surprise (33%), though the frequency of occurrence of these bark vocalisations are very low. Chuff vocalisations occurred mostly in the context of Anger (88%), as did growl vocalisations (89%), and growl-bark (100%), though the frequency of growl-bark is very low at just 1. Howl (the most well-known and studied of all wolf vocalisations, Dugnol et al. 2008; Harrington and Mech 1978; Harrington and Mech 1979; Harrington and Mech 1982; Nowak et al. 2007; Theberge and Falls 1967; Tooze et al. 1990) occurred only once (100%) in the context of Anxious. Whimper mostly occurred in situations where wolves were Anxious (61%) and Friendly (29%), and whine vocalisations were spread among events representing Joy (12%), Fear (18%), Anger (24%) and Friendly (41%), though Joy and Fear have very low frequencies of 2 and 3, respectively. Yelp mostly occurred in the context of Anger (67%) and yelp-bark occurs equally (50%) for both Anger and Fear, though yelp-bark vocalisations have very low frequencies for Anger and Fear at just 1 each. The low frequencies seen in Table S5 may indicate that the vocalisations used were

purely coincidental, but perhaps they are vocalisations that are used to emphasise the intensity of the affective state being conveyed (Le Roux 2002).

Table S6 shows that dogs use bark vocalisations mostly in the context of Anger (58%), Joy (33%) and Friendly (16%), which are the affective states that have the most facial movements associated with them (Table 4). However, bark vocalisations are also used to an extent in all affective states (except Interest). Chuff vocals (like wolves) occurred mostly in the context of Anger (100%) though with a somewhat low frequency of occurrence of 6. Growl also occurred mostly (like wolves) in the context of Anger (60%), and Joy (37%). Growl-bark (again, like wolves) mostly occurred with Anger (75%), though it does have a low frequency of 3. Grunt mostly occurred in the context of Friendly (50%) and Joy (38%), though both have low frequencies of 4 and 3 respectively. Whimper mostly occurred (just like wolves) in situations where dogs were Anxious (43%) and Friendly (35%), and to a lesser extent in the context of Joy (16%), which has a somewhat low frequency of 6. Whine vocalisations mostly occurred (again, just like wolves) in the context of Friendly (64%), and again, to a lesser extent with Anxious and Joy at 18% each. No howl, yelp or yelp-bark vocalisations were recorded.

No vocalisations were observed in the context of Curiosity in wolves (yet, they were for dogs, at very low frequencies, not exceeding 1), and no vocalisations were observed in the context of Interest in dogs (yet, they were for wolves, again at very low frequencies not exceeding 1). The observed limited vocalisations for Curiosity and Interest may be due to the fact that these affective states are essentially investigatory states and so, there is no need to vocalise when inspecting an object of curiosity or interest, as a vocal could alarm and cause evasion of that object (for example, if that object was potential prey).

Table S5. Frequency and percentage (in brackets) of occurrence of vocalisations per affective state observed in wolves, *n* = 137.

**Vocals**	**Affective State**								
	**Anger**	**Anxious**	**Curiosity**	**Fear**	**Friendly**	**Happy**	**Interest**	**Joy**	**Surprise**
**Bark**	0 (0)	2 (67)	0 (0)	0 (0)	0 (0)	0 (0)	0 (0)	0 (0)	1 (33)
**Chuff**	7 (88)	0 (0)	0 (0)	0 (0)	0 (0)	0 (0)	0 (0)	0 (0)	1 (13)
**Growl**	57 (89)	0 (0)	0 (0)	1 (2)	5 (8)	0 (0)	0 (0)	1 (2)	0 (0)
**Growl-Bark**	5 (100)	0 (0)	0 (0)	0 (0)	0 (0)	0 (0)	0 (0)	0 (0)	0 (0)
**Howl**	0 (0)	1 (100)	0 (0)	0 (0)	0 (0)	0 (0)	0 (0)	0 (0)	0 (0)
**Whimper**	0 (0)	17 (61)	0 (0)	1 (4)	8 (29)	0 (0)	1 (4)	0 (0)	1 (4)
**Whine**	4 (24)	0 (0)	0 (0)	3 (18)	7 (41)	0 (0)	1 (6)	2 (12)	0 (0)
**Yelp**	6 (67)	0 (0)	0 (0)	1 (11)	2 (22)	0 (0)	0 (0)	0 (0)	0 (0)
**Yelp-Bark**	1 (50)	0 (0)	0 (0)	1 (50)	0 (0)	0 (0)	0 (0)	0 (0)	0 (0)

Table S6. Frequency and percentage (in brackets) of occurrence of vocalisations per affective state observed in domestic dogs, *n* = 298.

**Vocals**	**Affective State**								
	**Anger**	**Anxious**	**Curiosity**	**Fear**	**Friendly**	**Happy**	**Interest**	**Joy**	**Surprise**
**Bark**	58 (41)	5 (4)	1 (1)	6 (4)	23 (16)	0 (0)	0 (0)	47 (33)	1 (1)
**Chuff**	6 (100)	0 (0)	0 (0)	0 (0)	0 (0)	0 (0)	0 (0)	0 (0)	0 (0)
**Growl**	34 (60)	0 (0)	0 (0)	2 (4)	0 (0)	0 (0)	0 (0)	21 (37)	0 (0)
**Growl-Bark**	3 (75)	0 (0)	0 (0)	0 (0)	0 (0)	0 (0)	0 (0)	1 (25)	0 (0)
**Grunt**	0 (0)	0 (0)	1 (13)	0 (0)	4 (50)	0 (0)	0 (0)	3 (38)	0 (0)
**Whimper**	0 (0)	16 (43)	1 (3)	1 (3)	13 (35)	0 (0)	0 (0)	6 (16)	0 (0)
**Whine**	0 (0)	8 (18)	0 (0)	0 (0)	29 (64)	0 (0)	0 (0)	8 (18)	0 (0)

Simulations to test the potential effect of individual or breed on the level of disagreement in our confusion matrix for dog affective states (Figure 3).

The level of disagreement in our confusion matrix for dog affective states (Figure 3) will to some extent be dependent upon the particular breeds of dog and individuals used, and the range of affective states expressed by each. Therefore, to examine the effect of individual and breed on the levels of disagreement between actual and predicted affective state in our confusion matrix for dogs we conducted the following simulations.

We iteratively re-ran our linear discriminant analysis (LDA) with the same data but removing each individual in turn. We then examined the overall level of agreement between actual and predicted affective state allocations in the resulting confusion matrices in comparison to the level of agreement reported for our LDA utilising the full data set (which was 65%).

These individual level simulations revealed that removing any one individual from the analysis resulted in a change of level of agreement (from the baseline of 65%) of between +1% to -57%. Thus, removal of some individuals resulted in levels of agreement dropping to as low as 8% overall. By contrast, the greatest increase in levels of agreement was minimal, to 66% (compared to the full LDA value of 65%). Thus, by including all of our individuals in the LDA we are in fact reporting a very conservative indication of overall levels of dis-agreement between actual and predicted affective states and maximising the potential level of agreement.

To test for breed effects we applied the same approach of re-running the LDA, but instead of removing single individuals, we removed all records for each breed in turn. These breed level simulations revealed the same effect; removing any one breed from the analysis resulted in a change of level of agreement (from the baseline of 65%) of between +2% to -56%. Thus, removal of some breeds resulted in levels of agreement dropping to as low as 9% overall. By contrast, the largest increase in levels of agreement was to 67% (compared to the full LDA value of 65%). Thus, again, by including all of the observed breeds in our LDA we are in fact reporting a very conservative indication of overall levels of dis-agreement between actual and predicted affective states and maximising the potential level of agreement. None of these simulations resulted in a substantial increase in levels of agreement compared to that reported in our manuscript.

These simulations demonstrated the same pattern of only increasing disagreement (and not increasing agreement) when the results for specific predicted and actual affective state combinations were examined, including the ‘Friendly’ state where we observed the majority of

disagreement in our reported LDA. Thus, estimates of disagreement between actual and predicted Friendly classifications within the confusion matrix, and the various 'incorrectly' predicted alternatives only increased on removal of any one individual or breed (**Table S7a and b**). So, removal of one or more individual or breed from our analyses would only serve to exacerbate the level of disagreement observed.

Table S7a: Summary results of LDA simulations removing each individual from the analysis in turn. 'Percentage in the full LDA' reports the percentage events that were incorrectly predicted by the LDA as 'Friendly' in relation to the 'Actual' affective state, as presented in Figure 3 in the main manuscript. The columns to the right of this provide summary statistics for the simulations, representing the degree of change in **disagreement** across all simulations for each combination of actual and incorrectly LDA predicted affective state involving 'Friendly'. No simulations resulted in a negative value, therefore no simulations resulted in an increase in agreement.

Affective state		Percentage in full LDA	Increase in **disagreement** in simulations				
Actual	Predicted		mean	median	min	max	se
Anger	Friendly	11.7	5.4	1.1	0.0	18.1	0.6
Anxious	Friendly	18.2	4.6	0.0	0.0	31.8	0.9
Curiosity	Friendly	13.6	13.0	13.6	0.0	27.3	0.7
Fear	Friendly	50.0	4.4	0.0	0.0	25.0	0.8
Happy	Friendly	53.3	11.7	13.3	0.0	20.0	0.5
Interest	Friendly	21.3	16.9	21.3	2.1	25.5	0.7
Joy	Friendly	25.5	6.2	1.9	0.5	17.8	0.7
Surprise	Friendly	16.7	4.8	0.0	0.0	20.0	0.8

Table S7b: Summary results of LDA simulations removing each breed from the analysis in turn. For details see Table S7a legend. Again, no simulations resulted in a negative value, therefore no simulations resulted in an increase in agreement.

Affective state		Percentage in full LDA	Increase in **disagreement** in simulations				
Actual	Predicted		mean	median	min	max	se
Anger	Friendly	11.7	5.4	1.2	0.0	18.3	1.0
Anxious	Friendly	18.2	4.5	0.0	0.0	18.2	1.0
Curiosity	Friendly	13.6	12.3	13.6	4.7	22.7	0.6
Fear	Friendly	50.0	5.1	0.0	0.0	25.0	1.4
Happy	Friendly	53.3	12.15	13.3	0.0	26.7	1.0
Interest	Friendly	21.3	16.47	17.8	4.3	23.8	1.0
Joy	Friendly	25.9	5.43	1.6	0.9	15.1	0.8
Surprise	Friendly	16.7	4.41	0.0	0.0	18.2	1.0

Table S8: Proportion of wolf and domestic dog events per primary, short-term, emotion-like affective state. Values depict number of events and corresponding percentage of events. See table S7a (supplied as a separate excel file; HOBKIRK-Table-S7a-BREED-BY-AFFECTIVE-STATE.xlsx) for details on dog breed-type per affective state (values show number of events, first percentage is percentage of events per row and second percentage is percentage of events per column).

	AFFECTIVE STATE									
Canid	Anger	Anxious	Curiosity	Fear	Friendly	Happy	Interest	Joy	Surprise	Total
Wolves	158 (28%)	22 (4%)	16 (3%)	69 (12%)	136 (24%)	32 (6%)	43 (8%)	39 (7%)	44 (8%)	559 (100%)
Dogs	94 (12%)	22 (3%)	22 (3%)	16 (2%)	289 (38%)	15 (2%)	47 (6%)	212 (28%)	36 (5%)	753 (100%)

References

Dugnol B, Fernandez C, Galiano G & Velasco J (2008). On a chirplet transform-based method applied to separating and counting wolf howls. *Signal Processing*, 88: 1817-1826.

Harrington FH & Mech LD (1978). Wolf vocalisations. In Wolf ad man evolution in parallel. Ed. Hall R & Sharp H, 1978, pp. 109-132. United States of America: *Academic press.*

Harrington FH & Mech LD (1979). Wolf Howling and Its Role in Territory Maintenance. *Behaviour*, 68: 207-249.

Harrington FH & Mech LD (1982). An Analysis of Howling Response Parameters Useful for Wolf Pack Censusing. *The Journal of Wildlife Management*, 46: 686-693.

Le Roux J (2002). Effective educators are culturally competent communicators. *Intercultural Education,* 13: 37-48.

Nowak S, Jedrzejewski W, Schmidt K, Theuerkauf J, Mysłajek RW & Jedrzejewska B (2007). Howling activity of free-ranging wolves (*Canis lupus*) in the Białowieża Primeval Forest and the Western Beskidy Mountains (Poland). *Journal of Ethology*, 25: 231-237.

Theberge JB & Falls JB (1967). Howling as a means of communication in timber wolves. *American Zoologist*, 7: 331-338.

Tooze ZJ, Harrington FH, and Fentress, JC (1990). Individually distinct vocalizations in timber wolves, (*Canis lupus*). *Animal Behaviour*, 40: 723-730.

Waller BM, Peirce K, Caeiro CC, Scheider L, Burrows AM, McCune S, & Kaminski J (2013). Paedomorphic facial expressions give dogs a selective advantage. PloS one, 8: e82686
